# RHOA at the intersection of inflammation-driven and sporadic colorectal cancer

**DOI:** 10.3389/fimmu.2026.1844372

**Published:** 2026-07-09

**Authors:** Sofia Elena Muccioli, Laura Hidalgo-García, Phuong A Ngo, Markus F Neurath, Rocío López-Posadas

**Affiliations:** 1 Department of Medicine 1, University Hospital Erlangen, Friedrich-Alexander-Universität Erlangen-Nürnberg, Erlangen, Germany; 2 Deutsches Zentrum Immuntherapie, Erlangen, Germany

**Keywords:** colitis-associated cancer (CAC), colorectal cancer (CRC), Cytoskeleton, Inflammation, RHOA

## Abstract

In the last decades, colorectal cancer (CRC) has emerged as a global problem, representing approximately 10% of all malignant tumors and the second leading cause of cancer-related death. Colitis-associated cancer (CAC) arises from chronic unresolved inflammation and is typically characterised by poor prognosis. Among key cellular regulators, the small GTPase RHOA controls cytoskeleton dynamics in various cell types and, thereby, key biological processes. Although it has been linked to various diseases, the role of RHOA in cancer remains controversial. RHOA has been proposed as a biomarker in cancer including CRC, but recent evidence shows that RHOA inactivation increases Wnt signalling and tumour risk in the murine intestinal epithelium. This review summarises the mechanisms of RHOA function and regulation and analyses the oncogenic pathways in which it is involved, with a focus on sporadic CRC and CAC. We discuss whether the RHOA pathway serves the same function in the two types of intestinal cancer and how the inflammatory context can modulate RHOA outcome following activation or inhibition, which still remains an open question. Finally, we address opportunities for therapeutic targeting of RHOA and related proteins. The understanding of the function of RHOA specifically in CRC may help identify limitations that might have hampered the development of novel targets for optimized treatment and/or diagnostic strategies.

## 1 Introduction

Latest data confirm that colorectal cancer (CRC) is the second leading cause of cancer death globally (1, 2). Notably, there has been a rising incidence of CRC among patients under 50 years of age in the last decades, also depicting high mortality rates. In patients with inflammatory bowel diseases (IBDs), the risk of developing CRC is increased compared to the general population, and the prognosis of IBD-associated CRC (colitis-associated cancer, CAC) is generally worse, with lower overall and cancer-specific survival than non-inflammatory CRC (or sporadic CRC) (3, 4). Furthermore, the literature highlights gaps in CAC research, particularly regarding predictive biomarkers, genomic alterations, and specific therapeutic targets (5–7). Interestingly, recent evidence highlights the partially overlapping mutation spectrum in sporadic CRC and CAC. Whole-exome sequencing in IBD-associated tumors demonstrated the existence of distinct genetic features in CAC and pointed to the relevance of cell communication, cell-cell signalling, and cell adhesion (8). Among those pathways, the central role of Rac and Rho GTPases subfamilies is further supported by their connection to the non-canonical Wnt pathway, as well as by the RHOA-mediated regulation of the cytoskeleton within Intestinal Epithelial Cells (IECs) and barrier function in IBD (8–10). In fact, recent evidence highlights the involvement of RHOA in both CRC and CAC (8, 11, 12), with studies showing that its function can be modulated differently depending on the biological contexts, such as the inflammatory microenvironment (13, 14) and tumor stage (13, 15). Actually, the role of RHOA in cancer is controversial, since it can act as both a tumour promoter and suppressor, depending on the specific cell types involved and the type of tumor (16). This manuscript aims at clarifying and contextualizing paradoxical observations about RHOA in cancer, focusing on CRC, and the dual role in sporadic CRC and CAC. We therefore provide an accurate and concise overview of current knowledge on this topic, discussing the potentially different biological contexts, like between sporadic and inflammation-associated tumours, and thereby highlighting the role of inflammation as a disease modifier in cancer. Finally, we discuss therapeutic opportunities targeting RHOA and its related proteins. Contextualizing the current knowledge about the role of this key regulator of cytoskeleton dynamics specifically for this tumor entity would hopefully pave the way for developing disease-specific markers and new approaches for therapy in the future, as well as identify limitations that might have hampered the development of novel targets for optimized treatment and/or diagnostic strategies.

## 2 RHOA in colorectal cancer

### 2.1 Colorectal cancer

CRC represents a global burden, particularly in developed countries. It accounts for approximately 10% of all diagnosed malignancies in the world and is the second leading cause of cancer death (2). Importantly, the percentage of young patients (<50 years old) presenting CRC increases every year (17). Clinical symptoms of CRC include a change in bowel habits, rectal bleeding, abdominal pain, weight loss, fatigue, iron deficiency, and anaemia — rather non-specific symptoms that make early diagnosis and, in general, clinical management difficult. Genetic background, ageing, dietary habits, lifestyle, environmental pollution, and the presence of other conditions, like IBD, are considered important risk factors for developing CRC; however, the exact aetiology is not yet fully understood.

Mechanistically, CRC primarily arises from two pathways (18, 19). The 70-90% of CRC cases follow the traditional adenoma-carcinoma sequence, characterised by the accumulation of mutations (APC, RAS, TP53) and chromosomal instability, resulting in microsatellite-stable tumours. In contrast, 10-20% arise through the serrated neoplasia pathway, which is linked to RAS and RAF mutations and epigenetic instability, leading to both microsatellite-stable and unstable cancers. In 2015, the CRC Subtyping Consortium identified four molecular CRC subtypes with distinct features, named consensus molecular subtypes (CMS) 1 to 4 (20). This classification aims to provide a basis for patient stratification and to guide treatment interventions.

Beyond the genetic and molecular heterogeneity of sporadic CRC, a clinically and biologically distinct entity must be considered: colitis-associated CRC (CAC). Although CAC constitutes a minor fraction of all identified CRC cases (around 2%), it is often associated with an unfavourable prognosis due to its treatment challenges (21–23). Unlike sporadic CRC, CAC is driven by sustained and unresolved inflammation that, through reactive oxygen and nitrogen species, promotes mutagenesis (24, 25). The genetic profile also differs: TP53 silencing usually takes place in early stages of CAC, while KRAS and APC mutations are less frequent and occur at later stages of tumour development (22). Using whole-genome sequencing, Robles et al. reported that genes such as PIK3CA, BRAF, SMAD4, and C-MYC are mutated in both sporadic CRC and CAC; however, the molecular alteration profile in CAC patients is distinct from that of sporadic CRC (8, 26). Notably, CAC-specific mutations were associated with Rho GTPase network, cell communication/adhesion, cytokine signalling, and chromatin remodelling, reflecting the key role of inflammatory mediators in the development of this tumour, and pointing towards the involvement of proteins governing cytoskeletal dynamics and intercellular contacts. Nevertheless, the attempts to generate knowledge to establish new biomarkers that help to distinguish CAC from sporadic CRC are limited in comparison to the well-studied genomic characteristics of CRC, justifying the need to further explore and develop this field (27, 28).

CRC can be managed by surgery or endoscopic resection along with medical treatments such as chemo-radiotherapy, biologics, immunotherapy, and when no conventional treatment has proven effective, rescue therapy. Generally, the chosen treatment depends on the stage of the disease at diagnosis. Where surgery is not possible, radiotherapy and chemotherapy are considered key strategies for CRC and metastatic CRC, respectively (29, 30). In patients with advanced CRC, the combination of chemotherapy with biologics - including EGFR inhibitors and anti-VEGF therapy - has been linked to prolonged survival and better quality of life (30, 31). Based on the CMS classification, several immunotherapies are now being considered for specific subtypes of CRC, including small molecules, macromolecules, adoptive cell therapy, cancer vaccines, and gene therapy (31). Moreover, advances in detection techniques — such as molecular endoscopy (32) — as well as the implementation of population-level screening programmes in healthcare systems, have contributed to the early detection of precursor lesions and, therefore, to improved clinical management.

Despite rapid advances in both screening and treatment, CRC continues to compromise patients’ quality of life. Although showing certain geographical heterogeneity, CRC mortality rates are projected to continue decreasing in most countries; however, the number of deaths is expected to reach 60% and 71.5% for colon and rectal cancer by 2035, respectively (33). Significant gaps also remain in our collective understanding of CAC carcinogenesis compared to sporadic CRC, and past and recent efforts aimed at discovering accessible biomarkers capable of distinguishing CAC from sporadic CRC have yet to close this gap (8, 34). Given the complexity of CRC and the need to tailor treatment to each patient, a thorough understanding of the molecular mechanisms underlying both sporadic CRC and CAC is essential.

### 2.2 Rho family proteins

Small GTPases are monomeric proteins that act as molecular switches, transmitting cellular signals through their enzymatic activity that hydrolyses GTP to GDP. They are involved in various cellular mechanisms: cytoskeletal dynamics, cell cycle progression, survival, proliferation, differentiation, trafficking, adhesion, and migration (35). Among a wide variety of small GTPases, the most studied are those belonging to the Ras superfamily, which in turn is divided into five families (Arf, Rab, Ran, Ras, Rho) (35, 36). Proteins within the Rho family (including well-studied RAC1, CDC42, and RHOA, among others) regulate F-actin and microtubule filament organization, and are therefore key players in cytoskeleton dynamics (37). Rho family proteins regulate fundamental biological processes such as cell morphology, cell migration, polarity, cell cycle, and vesicular transport (38). However, their activation can also favour aberrant situations. In fact, most small GTPases have been associated with pathology in general, and the development of cancer in particular (38). Different members of the Rho family, particularly CDC42, RAC1, and RHOA, are involved in multiple stages of cancer progression (39), underscoring their key role in this process.

#### 2.2.1 General concepts

In their cycling between GTP- and GDP-bound states (40), GTPases interact with three classes of regulators: guanine nucleotide dissociation inhibitors (GDIs), which retain them in the cytosol; guanine nucleotide exchange factors (GEFs), which facilitate GDP release; and GTPase-activating proteins (GAPs), promoting GTP hydrolysis, returning the protein to its inactive state (41). Rho family GTPases and their function fundamentally depend on the subcellular localization (42, 43). The Cys-Ali-Ali-X sequence (CAAX box) at the C-terminal hypervariable region is fundamental for the post-translational modifications occurring on Rho family proteins (44). Post-translational prenylation increases the hydrophobicity of the target protein by attaching isoprenoids synthesized via the mevalonate pathway to its structure (45, 46). Prenylated GTPases are taken over by a GDI (47) which acts as a molecular chaperone that transports them from the cytosol to the correct membrane, where they will then be activated by the RhoGEFs and interact with corresponding effector proteins (48). In the case of Rho family proteins, the key effectors are PAKs (p21-activated kinases), mDia (mammalian diaphanous-related formins), and ROCK (Rho-associated coiled-coil-containing protein kinase) (49–51). PAKs proteins are serine/threonine kinases downstream of RAC and CDC42 (52–55). In contrast, RHOA activates several structurally and functionally distinct downstream effectors, including ROCK1/2, mDia formins, PKN, CITRON kinase, and Rhotekin. Among these, the serine/threonine kinases ROCK1 and ROCK2 are closely homologous yet non-redundant: ROCK1 is primarily linked to apoptotic membrane blebbing and cell rounding, while ROCK2 is associated with actomyosin contractility, stress fibre formation, and cell migration (56, 57). In contrast, mDia formins promote actin polymerisation and assembly of unbranched actin filaments, producing cellular outcomes that are mechanistically distinct from those mediated by ROCK. Altogether, the pleiotropy of cellular functions regulated by Rho family proteins is made possible by certain functional overlapping between different family members, but also by a large number of downstream effectors and upstream regulatory mechanisms (58).

#### 2.2.2 Rho family members regulate cytoskeleton dynamics

RHO family GTPases regulate actin cytoskeleton remodelling, a strongly dynamic system to rapidly adapt to the changing needs of the cell (59). Hence, these proteins regulate a broad spectrum of cellular processes, such as control of cell shape, motility, cell growth and survival. Within the Ras superfamily, the Rho family consists of 20 members and can be divided into eight subfamilies: Cdc42, Rac, Rho, RhoBTB, RhoF, RhoH, RhoUV and Rnd. The Rho subfamily includes three very similar proteins, RHOA, RHOB, and RHOC, expressed in all tissues. In fact, RHOA/B/C interact in a different way with GAPs, GEFs, and GDIs, and their distinct functions within the cell are determined by their divergent subcellular localization and expression regulation (60). Thus, the presence of three isoforms in the Rho subfamily is only apparently redundant, as exemplified by the joint control of cell contraction during the last steps of cell motility, which depends on RHOA, RHOB, and RHOC (61).

### 2.3 RHOA and cancer

In contrast to the universal effect of mutations on Ras family proteins, the link between Rho family proteins and tumorigenesis is less well defined. For instance, RAC1 mutations are associated with specific types of cancer, such as melanoma (62) and head and neck cancers (63). In contrast, RHOA mutations are mainly associated with gastric carcinoma (64). Beyond this, alterations in RHOA function have been involved in several types of cancer, such as ovarian, breast, gastric, and colorectal cancer (65–67). Focusing on the Rho subfamily, the context of oncogenesis is where differences between RHOA, RHOB, and RHOC emerge the most. RHOA and RHOC are usually associated with cancer progression and metastasis, respectively, while RHOB seems to inhibit tumor growth (68, 69). Moreover, RHOA and RHOC have often been identified as cancer biomarkers, while RHOB is significantly downregulated in many cancer types (70–73). Despite several ambiguities concerning its role in cancer (see 2.6.1), RHOA displays a key task in several critical mechanisms during tumorigenesis.

#### 2.3.1 Cell proliferation

Transformation goes along with excessive proliferation, which enables tumor cells to outcompete normal cells for space and nutrients. In fact, cell cycle progression control appears among the pleiotropic functions of Rho family GTPases. In physiological situations, RHOA activity is particularly important during the last steps of mitosis, anaphase, and telophase; GTP-RHOA localizes at the plasma membrane and cleavage furrow, and promotes the contractile ring, permitting cytokinesis and the division of the cytoplasm for the two daughter cells (74). In addition, different evidence shows that RHOA is involved in promoting proliferation and cell cycle in the context of disease, including cancer and the dysfunctional proliferation of transformed cells. Actually, current knowledge supports the concept that both activation and inhibition of RHOA have been linked to promoted cell proliferation (75).

Regarding the involvement of RHOA in the promotion of CRC, the evidence is also conflicting. It has been shown that RHOA has a positive effect on cell proliferation in CRC (76), and inhibition of RHOA, and RHOC, through RNA interference reduced the growth of human colorectal xenografts in mice (11). For instance, RHOA promotes CRC proliferation through a serotonin-dependent upstream axis, where serotonylation and constitutive activation of RHOA lead to ROCK1/2-mediated YAP activation and nuclear localization. Importantly, inhibition of either RHOA or ROCK signalling markedly impairs the SERT–RHOA–ROCK–YAP axis involved in CRC cell proliferation and progression (76). Du et al. describe how HIF-1α (hypoxia-inducible factor 1α) activates the RHOA/ROCK2 signalling pathway to promote tumor growth and metastasis specifically under hypoxic conditions in the tumor microenvironment (77). In addition, inflammatory CXCL12/CXCR4 signalling in CAC upregulates RHOA to sustain tumor progression (14).

Notably, contradictory findings have also shown that RHOA inhibition may favour tumour cell proliferation. For instance, Rodrigues et al. described a mouse model characterized by APC mutations and the absence of Rhoa at the level of intestinal epithelium: these mice have an accelerated tumor development in the small intestine compared to wild-type mice (13). Importantly, an investigation of a cohort of CRC patients in this study revealed that RHOA is downregulated in metastases compared with primary tumors, suggesting a stage-dependent function, where loss of RHOA may facilitate the transition towards metastasis. In a further study, the same group showed that the occurrence of RHOA dominant negative mutations within IECs lead to Wnt-dependent spontaneous tumors in the murine small intestine. Mechanistically, lower RHOA activity led to enhanced proliferation and anchorage-independent growth due to the upregulation of Wnt signalling by Rho-associated protein kinase (ROCK) and diaphanous-related formin 1 (DIAPH1) (12). These controversial observations suggest that additional investigations are needed to fully clarify how proliferation is involved in RHOA’s function in the context of CRC, also taking into account the impact of inflammation.

#### 2.3.2 Cell shedding

During cell shedding, aged or damaged cells are extruded and replaced by healthier counterparts to maintain a constant cell number, allowing epithelial turnover and the physiological functions of the epithelial layer (78). Interruption or malfunction of this process can cause transient gaps in the gut lining (“leaky gut”) or formation of cell masses in the epithelial layer, potentially leading to inflammation or cancer, respectively. Therefore, cell shedding is considered a critical defense mechanism, and its dysregulation is often associated with infection, inflammation, and cancer (78–80).

During cell extrusion, both apoptotic and living shedding cells release S1P, which serves as a communication tool among the shed cell and its neighbours. S1P on the shedding cell’s surface activates S1P2 receptor of surrounding cells, and in turn, activates p115RhoGEF (a GEF for Rho subfamily GTPases) to foster RHOA-mediated actomyosin basolateral contraction and tight junctions/adherens junctions’ redistribution in those cells to drive apical extrusion of the shedding cell (81–83). In fact, inhibiting RHOA or Rho kinase entirely blocks both apoptotic and non-apoptotic cell shedding (83, 84). During physiological cell shedding, the highly coordinated rearrangement of tight junctions is necessary for preserving the integrity of the gut barrier. In fact, increased intestinal epithelial cell shedding, local barrier defects, and epithelial gaps have been detected frequently in IBD patients (80, 85, 86). In line with this, our previous data have shown that mice with impaired prenylation or Rhoa-deficiency in the intestinal epithelium exhibited defective cell shedding, along with a higher frequency for gap formation and disruption of the barrier’s function (9). These findings confirm the role of prenylated RHOA during cell shedding *in vivo* and suggest it as a promising target.

In the context of cancer, cell shedding acts as a tumor suppressor mechanism, which removes cells with oncogenic mutations from the tissue. This is known as EDAC, “Epithelial Defense Against Cancer” (87). EDAC is a form of cell competition: normal epithelial cells recognise and eliminate transformed and potentially cancerous cells by extruding them from the apical side (79). In this scenario, not only cell extrusion but also its direction must be tightly controlled to avoid these cells infiltrating underlying tissues, potentially contributing to metastasis (78, 81). In fact, frequently found mutations in cancer, including CRC, such as APC or KRAS, have been linked to the direction of cell extrusion (88). The cytoskeleton and mechanotransduction are central aspects in the functioning of EDAC: mechanical forces play key roles in the identification of transformed cells by healthy counterparts, while the cytoskeleton is essential to exert forces to eliminate aberrant cells on the apical side of the epithelium (50, 89, 90). Thus, RHOA is involved in both mechanotransduction and cytoskeletal functioning (91, 92). Of note, a truncation mutation on APC resulted in alterations of epithelial tension via the RHOA/ROCK pathway, impairing apical cell extrusion (93). In addition, mechanical tension contributes to the S1P effect, the leading to RHOA activation in neighbouring cells, which culminates in cell extrusion, further confirming the role of RHOA-mediated mechanotransduction in apical extrusion (83).

In the field of CRC, a recent study has shown that intestinal cancer cells with accumulated mutations exploit cell competition to enhance their own invasive ability. Thus, the additional activation of Wnt signals (β-catenin mutations) resulted in an impaired EDAC to extrude cells carrying Ras^v12^ mutations, which infiltrate diffusely into the tissue to form highly invasive cancerous tumors (94). Furthermore, two studies demonstrate that a cooperation between APC mutations and the secretion of WNT antagonists inhibits the proliferation of normal epithelial cells in the murine gut, defeating normal stem cells by diminishing their ability to replenish themselves and enabling tumor initiation (95, 96). Two other frequently detected mutations (KRAS and PIK3CA) also act on cell competition by releasing bone morphogenetic protein (BMP) ligands such as Bmp2 and Bmp7 and other factors that breakdown the crypt niche, resulting in remodelling and ultimately the elimination of normal surrounding cells (97). Notably, RHOA activation mediated by S1P2 was shown to participate in promoting the accumulation of filamin in the healthy neighbouring cells, which in turn promotes EDAC and the extrusion of mutated cells; and consequently, inhibition or knockdown of S1P2 prevents apical extrusion of neighbouring transformed cells (98). Similarly, mechanical forces generated within normal epithelial cells have been linked to the recognition and removal of transformed cells via immunological ligand-receptor interactions, such as leukocyte immunoglobulin-like receptor B3 (LILRB3) on normal cells detecting major histocompatibility complex class I (MHC class I) on transformed cells, which triggers the SHP2-ROCK2 pathway to induce extrusion (99). These studies highlight that both extrinsic signalling, through S1P-S1P2 and direct ligand-receptor recognition, converge on RHOA-mediated cytoskeletal remodelling to mechanically drive apical extrusion.

Overall, EDAC appears as a key mechanism for CRC initiation. Thus, RHOA-driven extrusion may support the elimination of transformed cells, and thereby act as a tumor suppressor during early stages of tumorigenesis. Accordingly, defects in apical cell extrusion, which is tightly controlled by S1P-RHOA signalling, emerge as a mechanism employed by cancer cells to escape the control of healthy counterparts, enabling them to form cell masses (tumor initiation) and invade tissues later (metastasis). Hence, targeting RHOA functionality may boost EDAC activity (extrusion of mutated cells) and prevent the early onset of CRC. However, the literature discussed in this section has predominantly described this mechanism in a colitis-independent context. Considering that chronic inflammation is widely recognised as a factor that increases the risk of developing CAC (100), subjacent inflammation due to RHOA deficiency within intestinal epithelial cells (9) might also modulate EDAC (92) and impact on early stages of CRC. Thus, the precise role of RHOA during tumor initiation and EDAC requires further investigation to better distinguish its contributions across colitis-associated and sporadic contexts, to better understand the functional link between defective epithelial RHOA and tumorigenesis.

#### 2.3.3 Cell migration, invasiveness and metastasis

A vital process for the successful spread of cancer is the epithelial to mesenchymal transition (EMT), which represents the transformation of immobile epithelial cells into cells with a mesenchymal phenotype, capable of moving (3). Although EMT is a gradual mechanism, its completion includes the disruption of cell-cell junctions, the loss of apical-basal polarity and the acquisition of front-rear polarity, and the detachment of the Focal Adhesions (FA) from the cellular matrix, which strictly depend on the cytoskeleton rearrangement. Furthermore, during cell migration, RHOA, RAC1, and CDC42 coordinate with downstream effectors to remodel the actin cytoskeleton, stabilize microtubules, and regulate membrane protrusions while acto-myosin contractility drives forward movement (101). Therefore, RHOA activation at the trailing end causes actin polymerization and force generation (69). Thus, it is not surprising that Rho family proteins also play a key role in EMT and metastasis (39). RHOA activation induces ROCK and mDia1-mediated modulation of actin polymerization and the dissociation of cellular junctions (102). Among those, E-cadherin downregulation is considered an early event during EMT, which has also been linked to metastasis (103). Controversially, RHOA, and also RAC1, are in charge of directing E-cadherin deposition into cell-cell junctions in epithelial cells (104, 105). In CRC, RHOA-LIMK2-cofilin-1 signalling controls cell detachment and migratory behaviour during EMT (106). Furthermore, through the control of actomyosin contractility and cell–cell junction stability, RHOA coordinates collective cell movement and preserves multicellular organization. Disruption of RHOA signalling impairs apical constriction, cell polarity, and junctional integrity, thereby altering collective migratory behaviour in epithelial cells (107), a process recognised as a major mechanism driving cancer metastasis.

Actually, while some studies identify RHOA as a factor that supports tumour cell migration and mechanical adaptation during cancer progression (75), other evidence suggests that RHOA may instead act as a metastasis suppressor. Hence, elevated RHOA expression has been associated with advanced tumor stages and increased lymphatic and vascular invasion, suggesting that its pro-tumorigenic role may become more prominent during later stages of disease progression (15). Similarly, other studies report that RHOA is upregulated in CRC tissues and, in conjunction with inflammation-related pathways such as STAT3 signalling, promotes cancer cell migration and invasion, further suggesting the role of RHOA within an inflammatory tumour microenvironment (108). In line with this, RHOA/ROCK pathway promotes metastasis and proliferation in the context of CRC, and its pharmacological inhibition reverses the EMT process (109). However, other evidence suggests that, particularly in the colon, RHOA may instead act as a metastasis suppressor, whose loss triggers more aggressive oncogenic signalling pathways, such as Wnt signalling (13). Similarly, clinical data indicate that reduced RHOA expression can predict recurrence and poorer survival in locally advanced CRC (Dukes’ C patients), further supporting its tumor-suppressive role in specific disease contexts (110).

There is still a heated debate about the positive or negative effects of RHOA on metastasis, as this protein can activate pleiotropic mechanisms with opposite outcomes. Furthermore, some studies have shown that RHOA can influence invasiveness and metastatic potential not only by acting directly on tumor cells but also by modulating the tumor microenvironment. For example, Kalpana et al. showed that the reduction of RHOA in tumor cells alters the composition of the tumor microenvironment (particularly fibroblasts and macrophages), thus promoting the formation of metastases (111). However, RHOA signalling activation within tumor cells upon contact with macrophages allowed the required cytoskeleton rearrangement for intravasation during metastases (112). Overall, these findings underscore the importance of tightly regulated spatiotemporal RHOA activity.

#### 2.3.4 Intracellular signalling

The behaviour of transformed cells and the occurrence of tumors is the result of a deregulated cellular machinery. CRC arises from the alteration of multiple signalling pathways, such as Wnt, PI3K/Akt, Hippo, and NF-κB (113). Actually, the Wnt pathway is indeed a clear example of a RHOA-regulated pathway in cancer. In non-canonical Wnt, small GTPases, including RHOA, function as effectors upon binding of Wnt ligands to receptors (114). Additionally, RHOA is associated with the canonical Wnt signalling pathway primarily through the regulation of β-catenin stabilisation and nuclear translocation. In particular, the ligand Wnt3A activates RHOA and ROCK, which, through GSK-3β phosphorylation, leads to β-catenin translocation to the nucleus, where it activates the transcription of target genes of the canonical Wnt pathway (115). Despite the link between RHOA activity and Wnt pathway activation, chronic inhibition of RHOA in the intestinal epithelium disrupts E-cadherin/β-catenin, induces persistent nuclear accumulation of β-catenin and enhances the activation of Wnt target genes, thereby promoting loss of differentiation and metastatic progression (12, 13). Thus, the assumption that both activation and inhibition of RHOA can promote tumorigenesis can also be translated to the outcome on Wnt activation, further emphasizing the need for more investigations in order to discriminate cofounders affecting the behaviour of RHOA within tumors. Seeking at an explanation for this dichotomy, Dopeso et al. described a mechanism by which Wnt pathway activation induces c-MYC expression, impairing the binding of SP1 to the RHOA promoter in colon cancer cells, leading to the downregulation of RHOA (116).

The Hippo pathway has a vital role during embryogenesis and in organ homeostasis, but can also be implicated in a wide variety of diseases when dysregulated (117). The major effector of the Hippo pathway, (Pan) Yap-associated protein (YAP), is upregulated in many different tumor types (118, 119). As mentioned above, recent findings support the existence of a SERT–RHOA–ROCK–YAP axis involved in CRC cell proliferation and progression (76).

MAPK signalling is broadly implicated in cancer proliferation and CRC progression, with even therapeutic potential (120). Experimental models support a functional RHOA–MAPK axis in regulating actomyosin contractility, cell adhesion, and tumour growth, highlighting RHOA as a potential, but challenging, therapeutic target in cancer and CRC (121, 122). Overall, the impact of RHOA on the progression of CRC appears to be strongly influenced by the molecular and cellular context, involving interactions with the Wnt, Hippo, and MAPK signalling pathways, which together can determine whether RHOA acts as a tumor-promoter or tumor-suppressor.

### 2.4 RHOA beyond epithelial cells

Rho family GTPases regulate essential cytoskeletal processes — including migration, adhesion, immune synapse formation, vesicular trafficking, and phagocytosis — that determine how immune cells detect, reach, and eliminate malignant cells. Among these, RHOA — together with RAC1 and CDC42 — has emerged as a particularly important regulator across multiple immune cell types, resulting in diverse and sometimes opposing roles, with direct consequences for anti-tumour immunity and immune escape.

In NK cells, regulation of F-actin is essential for various stages of cell-mediated cytotoxicity (123). This has been linked to CDC42 (124), RAC1 (125), or a combination of RAC1 and RHOA (126), as well as to shared GEFs (127), though a direct role for RHOA itself has not been established (128).

In Dendritic cells (DCs), RHOA regulates homeostatic proliferation, survival, and antigen presentation capacity (129, 130). In fact, in the context of cancer, VEGF impairs DCs migration through the VEGFR2-RHOA-cofilin1 pathway, suggesting that impaired motility of DCs by VEGF is one of the aspects of immune escape mechanisms of tumours (131).

Macrophages participate in the removal of cancerous cells via phagocytosis, directly contributing to the anti-tumour response. RHOA signalling via ROCK has been shown to influence the engulfment of apoptotic cells (132), while RAC1 and CDC42 may exert opposing effects (133). RHOA also shapes macrophage migratory behaviour: its inhibition accelerates motility but impairs matrix adhesion, and its depletion can reduce cell migration and phagocytic capacity (134). In the tumour context, the onset of macrophage mesenchymal migration, which promotes tumour development, depends on the Rho/ROCK pathway (135). In neutrophils, RHOA orchestrates cytoskeletal dynamics to guide directional migration, chemotaxis, and tissue infiltration. A PHD2-HIF2α-RHOA axis has further been shown to modulate neutrophil motility in confined environments (136), a condition frequently encountered in the tumour microenvironment, suggesting that RHOA activity may influence the ability of neutrophils to penetrate tumours and contribute to immune surveillance or tumour-promoting inflammation.

The association between RHOA and immunity extends to the adaptive immune response, where it equally shapes anti-tumour immunity. In T cells, RHOA coordinates integrin-dependent adhesion (137), mitochondrial fitness in thymocytes (138), TCR-induced activation (139), and differentiation pathways relevant to tumour immunity, including Th2 and Th17 lineages (140–142). Our own data have shown that RHOA plays a decisive role in T cell homing and pro-inflammatory features in the gut — an effect less prominent for RAC1 and CDC42 (143). With respect to cancer immunosurveillance specifically, Kalim et al. demonstrated that RHOA is crucial for maintaining regulatory T cells (Treg) homeostasis and fitness, and for balancing autoimmunity and anti-tumour T cell immunity (142). Similar to T lymphocytes, the generation of B cells and their progenitors depends on RHOA (144, 145). Vadakumchery et al. demonstrated that conditional RhoA-deficient mice suffer a complete block in pre-B cell differentiation and survival and develop increased levels of autoreactive antibodies.

Taken together, the evidence reviewed above demonstrates that RHOA and other Rho family GTPases exert critical and context-dependent roles across both innate and adaptive immune cells. However, despite these well-established functions, the specific contribution of RHOA to immune cell behaviour in the context of CRC remains largely unexplored — a gap explicitly recognised in the field (42). Furthermore, these findings indicate that any therapeutic strategy targeting RHOA or other Rho-GTPases in cancer must consider not only their autonomous effects on tumour cells, but also their diverse and sometimes opposing roles in immune cells. This duality further highlights why manipulation of RHOA can produce paradoxical results depending on whether the primary target is the tumour or the anti-tumour immune response/tumor immune escape mechanism. A deeper understanding of these context-dependent functions may not only help resolve the seemingly contradictory roles attributed to RHOA in cancer, but also provide a foundation for the development of more precise, personalised immunotherapeutic strategies tailored to the molecular and inflammatory context of each patient’s disease.

### 2.5 RHOA-related proteins and CRC

The link between RHOA and cancer also involves mutations in its regulators and effectors, which alter RHOA activity and thereby impact tumour development (**Table 1**). In CRC, YTHDF1 copy number gain promotes metastatic progression by enhancing ARHGEF2 translation and RHOA activation. Notably, ARHGEF2 silencing markedly reduced tumour growth and metastasis *in vivo* (148). In line with this, the expression of the GEF Tiam1 is significantly increased during EMT, promoting cytoskeletal reorganisation, invasiveness, and the migratory properties of colorectal cancer cells (147). Increased expression of p190RhoGEF has also been reported in CRC and linked to FA turnover and cell migration (149). In contrast, the RhoGEF LARG has been proposed as a tumour suppressor, as its downregulation correlates with genomic loss in colorectal and breast cancer (150).

**Table 1:** RHOAs, GEFs, GAPs, GDIs and effector proteins.

Regulator type	Name	CRC: type and specific feature	Implication due to RHOA interaction	References
GEFs	Tiam1	Sporadic CRC	Cytoskeletal reorganisation, invasiveness and cell migration	(146, 147)
	YTHDF1	CRC	Metastases	(148)
	p190RhoGEF	CRC	Cell migration	(149)
	LARG	CRC	Tumor suppressor	(150)
GAPs	DLC1	CRC (advanced stages)	Downregulation leads to cell proliferation, migration and metastases	(151)
	p190GAP/GAP5	• CAC • Metastatic CRC	• Cell cycle progression • EMT and invasion	(152) (153)
GDIs	Sumoylated GDI1	CRC	Metastasis	(154–156)
ROCK		CRC progression	Proliferation, migration and invasion, tumour microenvironment modulation	(157–159)

RhoGAPs are frequently mutated in cancer and often exert tumour suppressor functions, such as DLC1, which is downregulated in multiple carcinomas, facilitating tumour cell migration and invasion (160, 161). In CRC, DLC-1 is frequently silenced via promoter methylation, leading to a loss of its regulatory function. This downregulation is associated with advanced stages of CRC development, increased proliferation, cell migration and metastasis formation (146, 151). Taking advantage of a mouse model of colitis, Shimomura et al. compared the genetic mutational profiles of sporadic CRC vs. CAC using *Sleeping Beauty* transposon mutagenesis screening; this genome-wide technique allowed them to discover cancer driver genes promoting CAC. They found that the *Arhgap5* gene, which encodes for p190BRhoGAP, a negative regulator of Rho family GTPase activity, affects cell cycle progression in this context. Hence, loss of ARHGAP5 has been identified as a driver of CAC by affecting cell cycle progression, thereby showing that ARHGAP5 displays a protective role in inflammatory contexts (152). In contrast, in sporadic CRC patients, ARHGAP5 can promote EMT and invasion through negative regulation of RHOA (153). This demonstrates that the regulatory function of RHOA, via ARHGAP5, depends on the biological context of the tumour, and more specifically on the ongoing inflammation.

Altered expression of GDIs has also been linked to tumour progression (162). Both over- and under-expression of GDIs have been reported in several cancers, including CRC, where increased GDI1 levels correlate with metastasis and poor prognosis (154, 163, 164). Post-translational modifications further modulate GDI function: GDI1 sumoylation enhances RHOA binding and reduces cancer cell motility, whereas inhibition of this modification by XIAP decreases migration in CRC cells (155). Altogether, altered expression or activity of GEFs, GAPs and GDIs can influence the activation status of RHOA in CRC in different ways, thereby contributing to both pro-tumour and anti-tumour function, and importantly, can be influenced by the cellular context.

The functional diversity of RHOA signalling is also determined by the specificity of effectors, as different downstream effectors can mediate distinct and even opposing biological outcomes in cancer. Among the downstream effectors of RHOA, ROCK signalling appears to play a particularly prominent role in cancer progression and related cellular processes (165, 166). In CRC, the overexpression of ROCK has been postulated as a prognostic marker (157). Nevertheless, other RHOA effectors, including mDia, contribute to cytoskeletal organisation, cell polarity, migration and invasion as well. In fact, ROCK and mDia activation can result in different, if not opposite, effects, as it has been shown for the maintenance of adherens junction complexes (167). Therefore, the biological outcome of RHOA activation depends on the balance between multiple downstream pathways, and not only on ROCK signalling.

### 2.6 Exploiting RHOA function in the context of cancer

RHOA pathway is involved in malignancy and may influence the initiation, growth and invasiveness of cancer cells through complex mechanisms, compelling its regulation of cytoskeletal rearrangement. Although still challenging, exploiting RHOA and its related proteins for therapeutic purposes offers promising opportunities in cancer treatment. Numerous efforts are currently underway to develop viable therapeutic approaches, but most of them involve still preclinical studies.

#### 2.6.1 RHOA plays a dual role in cancer

A fundamental question in cancer concerns the dual role of RHOA as tumor promoter or tumor suppressor (**Table 2**). In breast and pancreatic cancer, RHOA overexpression has been associated with increased tumor cell proliferation and growth, likely through cytoskeletal rearrangement and proliferative programmes that enhance oncogenic signalling (180). In gastric cancer, RHOA promotes tumor progression through activation of the RHOA–ROCK–CDK4/6 axis, leading to enhanced cell-cycle progression and proliferation (181). In small cell lung cancer, RHOA activity supports tumor cell proliferation and survival, through Wnt-dependent mechanisms (158). In adult T-cell leukaemia/lymphoma (ATLL), activating mutations in RHOA (e.g., Cys16Arg) act as gain-of-function alterations that drive malignant transformation by rewiring downstream signalling networks (182). Similarly, in other B- and T-cell lymphomas, dysregulation of RHOA-dependent signalling contributes to aberrant lymphocyte activation and clonal expansion, promoting lymphomagenesis (183–185). Hence, RHOA acts as tumor promoter when it is involved in pro-oncogenic patterns that result as potentiated with its action. In contrast, RHOA can also act as a tumor suppressor through the stabilization of cellular architecture and tissue homeostasis, which avoids the escalation of oncogenic signalling. For example, in skin cancers, RHOA contributes to epithelial integrity and thereby limits malignant progression and invasive behaviour, then controlling tumor expansion (186). In the hematopoietic system, particularly in T- and B-cell compartments, RHOA maintains normal immune cell homeostasis. Its functional loss leads to dysregulated lymphocyte behaviour and predisposes to lymphoid malignancies (183, 185). In K-Ras–driven lung adenoma, loss of RHOA activity enhances tumor initiation and progression, indicating a suppressive role in early lung tumorigenesis, where RHOA appears to constrain oncogenic K-Ras signalling outputs (174).

**Table 2:** RHOA as a tumor promoter/suppressor in different cell types.

Cancer type	Tumor promoter or suppressor?	Cell type involved	Role of RHOA	References
Breast Cancer	Both	Epithelial cells	• Stabilizing cell junctions reduces metastasis • RHOA correlates with metastasis and progression	(168); (66); (169); (170)
Gastric cancer	Suppressor	Epithelial cells	• Reducing proliferation through ROCK and mDia	(171)
CRC	Both	Epithelial cells	• RHOA absence induces Wnt pathway activation and increased proliferation • Inducing metastasis and proliferation through the YAP/Hippo pathway	(12); (172); (106); (113); (76)
CAC	Both	Epithelial cells	• Inducing metastasis through CXCL12/CXCR4 and RhoA activation • RHOA absence alters shedding	(14); (42)
Gliomas	Promoter	Epithelial cells	Promoting cell cycle progression	(173)
Lung cancer	Promoter	Epithelial cells	Enhancing K-Ras	(174)
Hepatocellular Carcinoma	Promoter	Epithelial cells	Inducing metastasis formation	(175); (176)
Pancreatic cancer	Suppressor	Pancreatic acinar cells	RHOA absence increases invasiveness through the ROCK1-Limk1-Cofilin1 pathway	(177); (178); (43)
Synovial Hyperplasia	Promoter	Synoviocytes	Inducing proliferation	(179)

Taking together and trying to understand the context that might define the outcome of RHOA function in cancer, this duality is clearly exemplified by the comparison between epithelial and haematological cancers. Thus, RHOA mRNA and protein levels are elevated in intestinal tumours compared to normal colonic tissue, suggesting that RHOA overexpression could act as a tumor promoter in epithelial tumors (14). In contrast, when it comes to the immune system, its loss of function in T and B cells promotes the development of tumours such as Angioimmunoblastic T-cell, paediatric Burkitt’s, and Diffuse Large B-Cell lymphomas (DLBL) (183–185). These observations suggest that the differing roles of RHOA may reflect broader differences between tumour types arising from distinct cell lineages (187). Thus, developing mouse models with tissue-specific Rho family proteins mutations is critical for a deeper understanding of their role in specific cell and/or cancer types. For instance, our group demonstrated that RHOA dysfunction within IECs is a crucial aspect in the pathogenesis of IBD, since mouse models with specific deletion of *Rhoa* or *Pggt1b* in intestinal epithelial cells (IECs) develop spontaneous intestinal inflammation (9, 80).

Furthermore, distinct RHOA mutations can also explain opposite outcomes in cancer (188). For instance, RHOA acts as a tumour suppressor in the case of dominant-negative mutations, particularly in the G17V mutation found in T-cell lymphomas. In contrast, other mutations ultimately act as gain-of-function oncoproteins that drive tumour development in lymphomas and gastric cancer (189). These paradoxical effects have even been observed within the same cancer type. In adult T-cell leukaemia/lymphoma (ATLL), 15% of ATLL cases harbour recurrent gain-of-function mutations in the RHOA gene (Cys16Arg) (182). Interestingly, other activating (Cys16Arg and Ala161Pro) and dominant-negative (Gly17Val) mutations of RHOA have also been described (182), suggesting that different RHOA alterations may have distinct impacts on the development of ATLL. Thus, in haematological malignancies, different mutational profiles can determine whether the RHOA gene acts as a tumour promoter or a tumour suppressor.

Beyond the notion of different outcomes upon RHOA activation depending on the specific mutation or the targeted cell type or tissue, the capacity of RHOA to promote/hinder tumorigenesis depends on the cellular context. Zandvakili and colleagues demonstrate that murine K-Ras’s lung adenoma is promoted by the loss of RHOA activity (174). In contrast, RHOA knock-out or chemical perturbation inhibited small cell lung cancer proliferation, in a Wnt-dependent mechanism (158). Another example is presented by Carvalho Santos, whose recent review concluded that targeting RHOA as a therapeutic strategy in haematological cancers could have an unpredictable outcome, depending on the cellular context (75). Together, we can state that RHOA function can result in different, if not opposite, outcomes in cancer depending on the cell type, the organ, the mutational profile, and the cellular context.

Furthermore, RHOA can act as a tumor promoter or suppressor depending on which downstream effector it engages. For example, RHOA promotes proliferation via RHOA–ROCK–CDK4/6 signalling in gastric cancer cells, or tumour growth through ROCK activation and cyclin D1 upregulation in CRC (11, 190); while alternatively restricts or alters invasion dynamics through RHOA–mDia-dependent cytoskeletal regulation in glioblastoma (75, 181). Of note, the occurrence of mutations on RHOA can result in protein conformational changes, which might also affect the interaction with downstream effector proteins. In gastric cancer, Y42C is considered an activating mutation engaging ROCK-dependent FAK and Hippo pathway, and thereby contributing to cancer initiation and metastasis (191). In contrast, L57V is also a gain-of-function mutation, but in this case, Hippo activation is due to GF1R and PAK1 as atypical effector proteins (192).

This dichotomy concerning RHOA in cancer is also relevant in gastrointestinal tumors. Old studies have suggested an upregulation of RHOA within CRC tumors (190), and considered it an oncogene in CRC (15, 77, 193, 194). Consistently, Zhang et al. showed that RHOA activation and STAT3 phosphorylation are increased in CRC and cooperate in promoting tumor cell invasion and migration, while their knockdown significantly reduces these abilities (108). More recent studies have challenged the assumption of oncogenic RHOA and support that both activation and inhibition of RHOA can trigger and/or contribute to tumorigenesis in the intestine. In fact, RHOA inhibition (via RNA interference) and RHOA deletion contributed to the initiation and progression of experimental tumorigenesis (11–13), and Muccioli et al. similarly reported that the loss of epithelial Rhoa induces chronic intestinal inflammation and spontaneous colon carcinogenesis (195). In addition, recent evidence shows that the upregulation of small GTPases such as RHOA and RAC1 in tumor tissue is strongly influenced by the mechanical tension within the tumor, making their use as reliable biomarkers even more challenging (193). The studies involving CRC in which RHOA appears predominantly oncogenic describe an advanced, invasive, hypoxic or inflammatory tumor setting. Yu, Qu et al. describe how RHOA is involved in proliferation and progression and how inhibition of RHOA or ROCK markedly impairs the SERT–RHOA–ROCK–YAP axis involved in this (76). In patients with established CRC, RHOA was highly expressed, and its knockdown in metastatic cell lines reduced migration and invasion (15). Similarly, in another study, the RHOA/ROCK pathway promoted metastasis and proliferation of CRC, and its pharmacological inhibition reverses CRC liver metastasis (109). Consistently, Zhang et al. showed that RHOA activation and STAT3 phosphorylation are increased in CRC and cooperate in promoting tumor cell invasion and migration, while their knockdown significantly reduces these abilities (108). This last study links the feature of invasive CRC tumors to another important feature associated with the tumor-promoting role of RHOA: inflammation. In fact, it was demonstrated that the CXCL12/CXCR4 inflammatory signalling pathway upregulates RHOA and promotes tumour progression in CAC, directly linking inflammation and the pro-tumoral role of RHOA (14). In parallel, Du et al. demonstrated that HIF-1α activates the RHOA/ROCK2 signalling pathway under hypoxic conditions, thereby promoting tumour growth and metastasis in CRC (77). In synthesis, these studies connect advanced tumor stage and metastasis, inflammation, and ROCK signalling to a tumor-promoting role of RHOA.

On the other hand, early tumorigenesis, epithelial barrier integrity, EDAC, and Wnt signalling pathway are all aspects associated with a tumor-suppressive role of RHOA. Rodrigues et al. describe an early tumorigenesis in an APC-mutated mouse model in which RHOA deletion promotes tumorigenesis (13). Ten years later, the same research group demonstrated that RHOA inhibition in murine intestinal epithelium promotes Wnt-driven spontaneous intestinal tumorigenesis (12). Accordingly, our group highlighted how RHOA is protective for epithelial integrity, since mice lacking RHOA in IECs show IBD-like symptoms as a defective barrier, altered cell shedding, and inflammation, features that point towards the direction of a pre-neoplastic inflammatory setting (9, 195). As explained above, EDAC functionality was associated with RHOA, which can prevent the early onset of CRC, therefore acting as a tumour suppressor. This is supported by the study of Gan et al., for example, where it was described how an APC mutation resulted in alterations of epithelial tension via the RHOA/ROCK pathway, impairing apical cell extrusion (93), and by Yamamoto et al., who performed an *in vitro* study on epithelial cells demonstrating how RHOA contributes to EDAC and tumoral cell extrusion (98). In conclusion, the RHOA tumor suppressive effect appears during early tumor initiation, and is likely related to the regulation of epithelial homeostasis.

Another emerging aspect is that different regulators may activate distinct spatial and functional pools of RHOA, influencing its behaviour as tumor promoter/suppressor. Various GEFs, such as ARHGEF2, Tiam1, and p190RHOGEF, emerged as associated with a pro-tumoral role, while LARG was associated with a tumor suppressive role of RHOA in CRC (147–150). It is interesting to notice how only LARG among other GEFs has been proposed as a tumour suppressor; in fact, its downregulation correlates with genomic loss in colorectal and breast cancer (150). This evidence suggests and reinforces the idea that RHOA’s role is strongly context-dependent: its activation through GEFs doesn’t always lead to the same final outcome. Strongly aligned with this, also ARHGAP5 led to both tumor promotion and tumor suppression, and this example reinforces how the biological context determines the final outcome of RHOA signalling, since on one side ARHGAP5 was identified as protective in a CAC mouse model, while it acted as pro-tumorigenic and pro-invasive in CRC cell lines (152, 153).

Overall, RHOA exhibits a consistent, context-dependent dual role in tumor development and across all tumour types. This duality is still under investigation, since mutations of RHOA can alter its interaction with the effectors and generate distinct effects (16). Moreover, the multiple effects of RHOA stem from the diversity of its regulatory mechanisms and downstream signalling interactions, combined with the partial functional redundancy among the proteins of the Rho family. Furthermore, several studies indicate that these interactions depend on the cellular context and external factors, such as inflammation, and additional genetic and epigenetic factors within the tumor. Summing up, RHOA, acting as a tumour promoter when involved in inflammatory, hypoxic or proliferative signalling networks (e.g., ROCK-driven pathways, Wnt, STAT3 or gain-of-function mutations), and as a tumour suppressor when it contributes to the maintenance of epithelial integrity, immune homeostasis or the early control of oncogenic transformation. A similar pattern is observed in CRC, where RHOA promotes invasion and metastasis in advanced tumours, but inhibits early tumorigenesis and supports epithelial barrier function.

**Figure 1:**
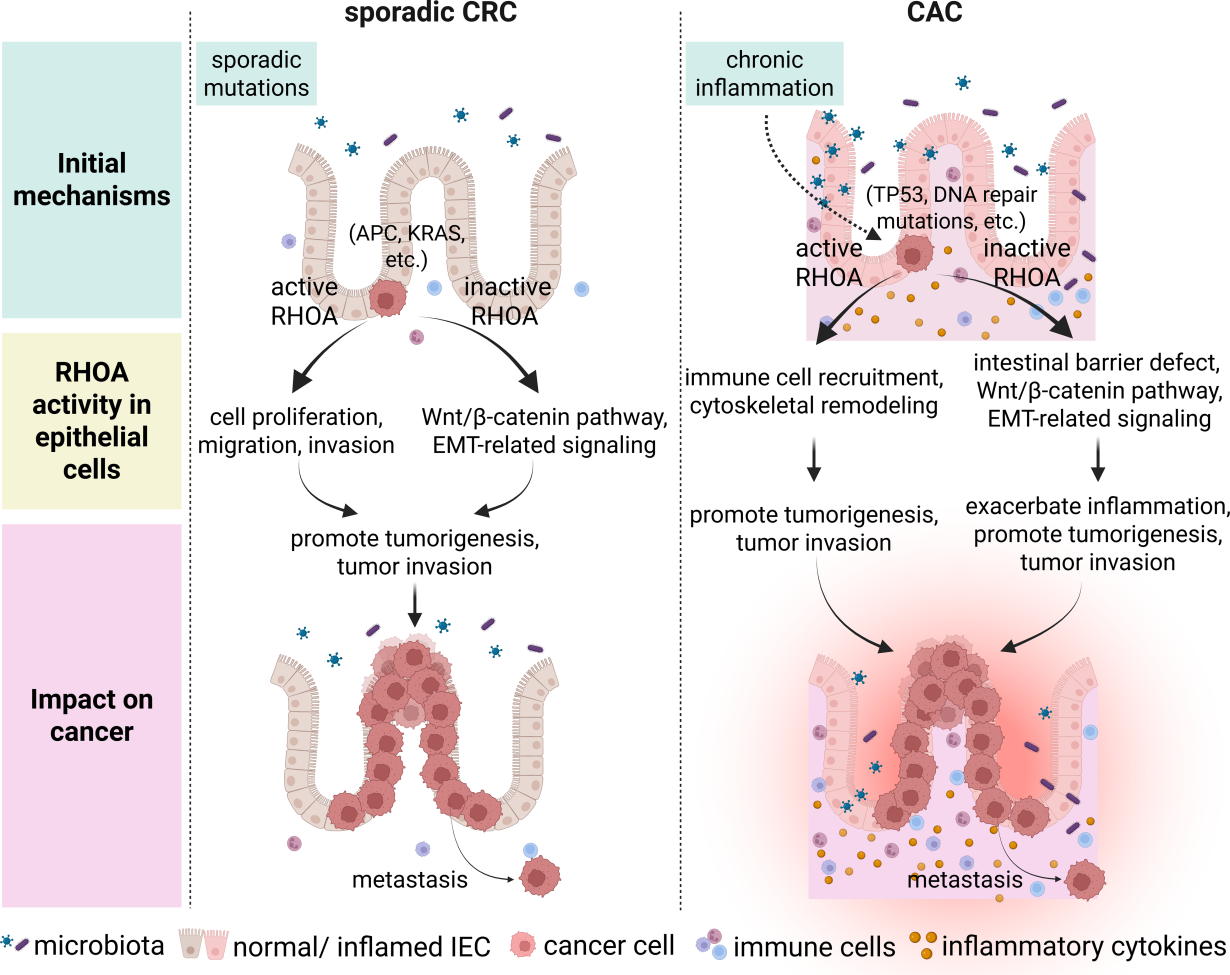
Multifaceted roles of RHOA in CRC. In sCRC, RHOA expression is linked to tumor invasion and poor prognosis, while its active form promotes cell proliferation, migration, and invasion (15, 153). Conversely, RHOA inactivation enhances Wnt/β-catenin and epithelial-to-mesenchymal transition (EMT)-related signaling, driving tumorigenesis and metastasis (13). In CAC, RHOA activity promotes EMT and immune cell infiltration, contributing to inflammation-driven tumorigenesis (14). Meanwhile, RHOA inactivation leads to intestinal barrier dysfunction (9), enhanced Wnt/β-catenin and EMT-related signaling, and could promote tumorigenesis and invasion (195). Created in BioRender. Ngo, P. (2026) https://BioRender.com/44r81yo.

In intestinal tumors, it is important to consider how inflammation might determine the outcome of RHOA activation/inhibition, as it remains unclear whether the functional behaviour of the RHOA pathway is conserved between sporadic CRC and CAC. Of note, findings in the last decade point to an association between small GTPases and CAC. Strikingly, Robles et al. demonstrated that 50% of IBD-related tumours harbour at least one mutation in genes associated with the small GTPases RHO and RAC (8). Additionally, Dregelies et al. have recently identified KRAS G12D as a specific mutation in CAC versus sporadic CRC, as well as mutations in ATM differentiating conventional from non-conventional CACs - a distinction referring to CACs arising in the context of IBD with or without prior dysplasia, respectively (34). Indeed, distinct clinical and molecular profiles in sporadic CRC and CAC, the latter being driven primarily by the long-term, persistent tissue lesion caused by inflammation, might also influence how Rho family GTPase-mediated signalling contributes to tumorigenesis (**Figure 1**). A representative example is provided by Yu et al., who demonstrated that activation of the CXCL12/CXCR4 axis promotes invasion and metastasis in CAC through upregulation of RHOA expression (14). Thus, CXCR4 contributed to the recruitment of immune cells and cytoskeleton remodelling through the lncRNA XIST/miR-133a-3p/RHOA axis. CXCR4 and RHOA mRNA and protein levels were significantly higher in human CAC than in normal colon tissue. Notably, the inflammatory and hypoxic signals present in the tumour microenvironment can further potentiate RHOA-driven tumorigenic pathways in CRC. Du et al. demonstrated that HIF-1α activates the RHOA/ROCK2 signalling pathway under hypoxic conditions, thereby promoting tumour growth and metastasis in CRC (77). Since hypoxia is a common feature not only of solid tumours but also of chronically inflamed tissues, these findings suggest that inflammation and hypoxia may converge on RHOA signalling to support tumour progression. In the context of sporadic CRC versus CAC, one should also mention EDAC. Inflammation has recently been postulated as another factor (besides the presence of super competitors) that can contribute to EDAC failure at very early stages. Sustained inflammation can suppress apical extrusion of Rasv12-transformed cells in the mouse, subsequently promoting the growth of transformed cells in small intestinal and pancreatic epithelium (196, 197). This exemplifies a situation where the same mutation can lead to cancer, or not, depending on the cellular context. In this case, the pro-inflammatory milieu is changing the fate of the tumor cells, revealing new insights into the mechanism by which inflammation supports tumor initiation by diminishing EDAC at a very early stage. Thus, inflammation-mediated impact on EDAC could potentially explain the opposite behaviour of RHOA as a tumor-suppressor and tumor-promoter depending on the cellular context.

However, drawing a mechanistic distinction between sporadic CRC and in CAC is challenging, and further studies are needed to directly assess whether inflammation affects cell competition during tumor initiation. More in particular, it would be interesting to investigate if inflammatory pathways already implicated in CAC, including CXCL12/CXCR4 signalling, STAT3 activation, Wnt signalling and neutrophil infiltration, converge on the S1P-S1P2-RHOA axis to modulate EDAC efficiency and the directionality of transformed cell elimination.

#### 2.6.2 Challenges in Rho family proteins research

Typically, altered expression - and not only the mutation of Rho family proteins - leads to disease. Therefore, strategies are required to modulate the function of Rho family proteins by targeting GEFs, GDIs, and GAPs or effectors (198). However, this strategy is intricate because of the overlap between the regulators, leading to redundancy within the system, where there is a complex network of interrelated pathways. The functional outcome of the activation of a specific GTPase might be controlled by various GEFs, while a single GEF can control the activation of various proteins (199). Muller et al. justify the redundancy of GAPs and GEFs through a “regulator-centric” model: according to this, the regulators would be responsible for the spatiotemporal control of RHO activation and the downstream signalling events associated with them (200). A further important aspect contributing to the functional divergence among Rho family GTPases is the specificity of their effectors, which play a decisive role in shaping cellular responses (58).

There are some difficulties in translating information from basic research on Rho family GTPases into a clinical context. First, designing molecules that selectively target a single Rho family protein is challenging because the highly conserved GTP-binding domain increases the chances of simultaneously inhibiting multiple family members. Second, even with a successful modulator design, downstream effectors and regulators are often shared, and the pathways in which they are involved overlap. This makes it difficult to predict the outcome of an attempt to regulate a specific Rho family GTPase. Finally, regulation is highly dependent on cell type (or tissue) and cellular contexts. This adds a further layer of complexity to predicting the outcome of the modulation.

#### 2.6.3 Targeting RHOA in cancer - current approaches and limitations

Considering their association with a range of diseases, Rho-GTPases and their immediate downstream effector kinases are prime targets for pharmacological modulation of cell cytoskeletal dynamics. The vast majority of pharmaceutical developments targeting the cytoskeleton, and the Rho-GTPases specifically, have thus far focused on inhibiting proliferation in various malignancies, and there are several preclinical therapeutic strategies targeting the malfunction of RHOA in cancer. Direct pharmacological targeting of RHOA has historically been considered unfeasible due to its picomolar affinity for GTP and the absence of stable allosteric pockets on its surface (201). However, the development of structure-guided inhibitors targeting the RHOA-GEF interaction interface (202, 203), the discovery of a conserved cryptic allosteric pocket in the switch II region (204), and the emergence of PROTAC-based degrader (205) are collectively challenging this paradigm (206) and highlight the possibility of discovering new medical compounds targeting Rho GTPases.

Current approaches can be classified into two broad categories: i) direct RHOA inhibitors, which bind to RHOA itself to disrupt its activation or effector engagement, including inhibitors of the RHOA-GEF interaction interface; ii) indirect or non-specific RHOA inhibitors, including compounds that target RHOA-activating GEFs directly, prenylation, or downstream RHOA effectors (**Table 3**).

**Table 3:** Targeting RHOA in cancer.

Modulator	Mechanism	Pathology/model	Development/testing	Reference
Direct RHOA inhibitors				
Rhosin	• Inhibits RHOA/RHOC-GEF interaction • Reduces cell motility and invasion	• Breast cancer and melanoma (cell lines) • Lung metastasis in mice	Preclinical (*in vitro* & *in vivo* on mouse)	(202); (207)
Y16	• Binds RhoGEF LARG, impairing RHOA activation; • Can act synergistically with Rhosin	MCF7 breast cancer cells	Preclinical (*in vitro*)	(203)
JK compounds (JK-122, JK-136, JK-139, JK-206, JK-312)	• Inhibit RHOA • Reduce cell viability, migration and invasion	• Gastric cancer cell lines • Mouse xenografts	Preclinical (*in vitro* & *in vivo* on mouse)	(208); (209); (210)
CCG-1423 & CCG-203971	Inhibit RHOA-mediated MKL1/SRF and MRTF/SRF signalling reducing cell migration	Various cancer cell lines	Preclinical (*in vitro*)	(211); (212); (213)
Non-direct RHOA inhibitors				
ROCK-Inhibitors				
Y-27632	• ROCK1/2 • inhibitor • Competes with ATP at the catalytic site • Reduces MLC phosphorylation	Various cancer cell lines (hepatic, colorectal, HeLa…)	Preclinical	(214); (215)
Fasudil (HA-1077)	• Non-selective ROCK inhibitor • Impairs different processes	• Rabbit ocular hypertensive models • Neuronal cell cultures • Rat model of chronic hypertension	Preclinical (*in vivo*)	(216); (217); (218)
H1152	• ROCK inhibitor • reduces metastasis	• Melanoma xenograft models	Preclinical (*in vivo*)	(219)
AT13148	• Dual ROCK-AKT inhibitor • Blocks downstream RHOA/C signalling	Solid tumors	Phase I clinical trials	(220)
mDia targeting compounds				
Intramimics (IMM-01, IMM-02)	• mDia agonists • Trigger actin assembly, cell cycle arrest, apoptosis	• Colon cancer xenografts • Zebrafish embryos	Preclinical (*in vitro* & *in vivo*)	(221, 222)
Non-specific RHOA inhibitors				
Prenyltransferase inhibitors (FTIs, GGTIs)	• Block protein prenylation • Impair membrane localization of GTPases	Various cancers (colon, gastric, metastases…)	Preclinical & clinical	(223)
Statins	• Inhibit HMG-CoA reductase → reduce GGPP/FPP → inhibit RHOA prenylation • Effect: anti-proliferative, anti-invasive	• Colorectal cancer • Hepatocellular carcinoma • Breast cancer • Multiple myeloma • Paediatric brain tumors	Preclinical (*in vitro*, *in vivo*) & multiple clinical trials	(224); (225–227); (228); (229)

##### 2.6.3.1 Direct RHOA inhibitors

Rhosin/G04 is a RHOA- and RHOC-selective inhibitor binding to the surface region sandwiching Trp58 of RHOA, thereby impairing interaction with GEFs such as DBL, p115RhoGEF and PDZ-RhoGEF (202). Because it blocks GEF-mediated RHOA activation rather than the RHOA-effector interface, it is considered more stimulus-selective than ROCK inhibitors (202). Functionally, treatment with Rhosin/G04 has been shown to inhibit the motility and invasiveness of breast cancer cell lines *in vitro*, decrease lung metastasis in mice, diminish tumour cell adhesion, and impair cell migration and invasion in melanoma and breast cancer models (202, 207). Despite promising results, Rhosin/G04 or derivative molecules remain in preclinical stages due to high concentrations needed and pharmacokinetic limitations.

JK compounds are RHOA-binding compounds derived from the Rhosin/G04 hydrazide scaffold targeting the Trp58 site to modify RHOA-GEF interactions (208, 210). These compounds have shown to potently inhibit the viability, migration, and invasion of gastric cancer cell lines and suppress tumour growth in xenografts (208–210). However, up to date whether JK compounds preserve effector binding and exhibit GEF selectivity analogous to Rhosin/G04, or carry additional off-target interactions, has not been systematically characterised.

##### 2.6.3.2 Indirect or non-specific RHOA Inhibitors

Y16 is a compound that binds to the DH-PH domain junction of the GEF LARG, selectively impairing RHOA activation in breast cancer cells and acting synergistically with Rhosin/G04 (203). Due to the structural conservation of the DH-PH domain junction across the RGS-RhoGEF subfamily (LARG, PDZ-RhoGEF and p115RhoGEF), Y16 also inhibits RHOA interaction with the latter two members. However, it does not affect RHOA interaction with DBL-family members lacking this domain (DBL or LBC) (203).

CCG-1423 blocks RHOA/C-dependent MRTF-A/SRF transcriptional activity and reduces migration in several tumour cell lines *in vitro* (211, 230, 231). However, its efficacy is context-dependent: in glioblastoma cells, resistance to its anti-migratory effects has been reported (232). Moreover, its therapeutic potential is limited by cytotoxicity, which prompted the development of the less toxic and more potent analogue CCG-203971 (233). This compound retains the ability to suppress MRTF-A/SRF-driven transcription and cell migration (212, 213, 233), but similarly, its application in oncology remains confined to preclinical research, with no clinical trials initiated to date.

A critical consideration when evaluating upstream RHOA inhibition is the functional diversity of the RHOA effector landscape, as previously addressed in section 3.2.1. This functional divergence means that the net effect of upstream RHOA inhibition may be difficult to predict and likely to be context-dependent across cell types and tumour microenvironments. As Prudnikova et al. and Clayton and Ridley have argued, targeting effectors that are specific to a more limited subset of Rho GTPases may therefore represent a more tractable therapeutic strategy than inhibiting RHOA itself (56, 57).

The most widely used pharmacological tools for interrogating RHOA-ROCK signalling are the ATP-competitive ROCK inhibitors Y-27632, Y-33075, and fasudil (HA-1077). Y-27632 and Y-33075 block ROCK1/2 activity and downstream MLC phosphorylation across multiple cell types (214), with anti-invasive effects reported in bladder and tongue squamous cell carcinoma, among other cancer models (234, 235). Fasudil similarly inhibits ROCK1/2 and has been used in the treatment of cerebral vasospasm, making it the most clinically advanced compound in this class (236). H1152 is a further, more potent ROCK inhibitor, and has been reported to reduce lung metastases in melanoma xenografts through overexpression of membrane-bound FasL (219). Nevertheless, its application remains largely preclinical and it has not been approved for clinical use. However, all three compounds carry limitations due to their lack of selectivity. As ATP-competitive inhibitors, Y-27632, Y-33075, and fasudil block ROCK1 and ROCK2 simultaneously, precluding the dissection of isoform-specific contributions. While Y-27632 shows greater than 200-fold selectivity over PKA and PKC in cell-free assays, it retains meaningful off-target activity against MRCK — a downstream effector of CDC42 — at approximately 10-fold reduced potency relative to ROCK (237, 238). A 2023 study further demonstrated that Y-27632 acts through additional, as yet unidentified mechanisms beyond ROCK inhibition (238), complicating the exclusive attribution of its cellular effects to RHOA-ROCK signalling. Fasudil carries a substantially broader off-target profile, with only 3-fold selectivity over PKA and documented inhibitory activity against PKC, PKG, and MRCK (237, 239). This broader promiscuity is particularly consequential in cancer biology, where ROCK and MRCK play distinct but partially compensatory roles in actomyosin-mediated migration and invasion (39, 240). These off-target complications are further illustrated by paradoxical pro-invasive effects observed in CRC models. In SW620 human colon cancer cells, Y-27632 induced a pro-migratory phenotype and increased invasiveness in both 2D and 3D assays - an effect replicated by fasudil and by siRNA-mediated knockdown of ROCK1 or ROCK2 (241). Separately, ROCK2 inhibition has been shown to trigger collective invasion of colorectal adenocarcinoma by inducing leader/follower cell polarisation through a distinct mechanism (242). Together, these findings illustrate how off-target effects and context-dependent signalling can produce clinically undesirable outcomes with ROCK inhibitors in specific tumour types, and underscore the case for greater effector selectivity. A conceptually distinct approach is represented by AT13148, a dual ROCK-AKT inhibitor that has advanced to phase I clinical trials for solid tumours (220).

Beyond ROCK inhibitors, the mDia agonists IMM-01 and IMM-02 (intramimics) promote actin assembly and trigger growth arrest and apoptosis in colon cancer cells, reducing tumour growth in xenograft models (221). Their comparatively low toxicity relative to formin antagonists suggests that mDia activation may represent a promising therapeutic avenue (222). The contrasting outcomes produced by ROCK inhibition versus mDia activation in related cellular contexts reinforce the argument of Prudnikova et al. and Clayton and Ridley: because distinct effectors not only produce different biological outputs but can produce opposing ones within the same pathway, effector-selective targeting — rather than upstream RHOA inhibition — may be necessary to achieve the desired therapeutic effect while minimising toxicity.

Inhibition of prenylation enzymes represents an indirect means of suppressing Rho GTPase activity. Prenyltransferase inhibitors include farnesyltransferase inhibitors (FTIs), which have been primarily used to inhibit RAS-related proteins (26, 243), and geranylgeranyltransferase inhibitors (GGTIs), which are considered more relevant for Rho GTPases, though their lack of specificity complicates therapeutic application (45, 244, 245). Statins act further upstream, inhibiting HMG-CoA reductase — the rate-limiting enzyme of the mevalonate pathway — and thereby impairing isoprenoid synthesis more broadly (246). Through this mechanism, statins exhibit proapoptotic, anti-proliferative, and anti-invasive properties in cancer models (246–248). For example, lovastatin treatment in APC^min^ mice reduced tumour burden through suppression of RHOA, which in turn repressed Wnt and YAP/TAZ signalling in CRC (249). Statins have been explored in multiple clinical trials across cancer types, including acute myeloid leukaemia, paediatric brainstem tumours, hepatocellular carcinoma, CRC, multiple myeloma, and breast cancer (227, 228). However, further studies are needed to establish optimal dosing and identify synergistic combinations with other antitumour agents (229).

#### 2.6.4 RHOA and resistance to anti-tumor therapy

Another key consideration in the clinical management of cancer is the emerging role of RHOA in driving resistance to anti-tumour therapies. The earliest mechanistic evidence came from CRC models, where RHOA silencing was shown to reverse chemotherapy resistance through two complementary mechanisms: impairment of MRP3-mediated drug efflux via an NF-κB–iNOS–nitric oxide axis in doxorubicin-resistant cells (HT29-dx) (250), and broad multidrug resensitization in irinotecan-resistant cells through simultaneous downregulation of efflux transporters, restoration of pro-apoptotic signalling, and reduction of cancer stem-like cell populations (251). Together, these studies established RHOA overexpression as a potential marker of therapy resistance in CRC, and demonstrated that its inhibition could serve as a broad chemosensitisation strategy.

This principle has since been confirmed across a wide range of cancer types and treatment modalities. Later, Adua and colleagues show that RHOA promotes drug resistance and metastasis-associated gene expression during Osimertinib treatment, promoting the development of residual EGFR-mutant cancer lung cells in the brain and accelerating the growth of CNS metastases (252). A similar effect has been observed in prostate cancer and enzalutamide resistance (253), dual BRAF/MEK inhibition in melanoma (254), temozolide in glioblastoma (173), or regorafenib in hepatocellular carcinoma (255). But the protective role of RHOA inhibition is not restricted to chemotherapy, and an equivalent mechanism could be described for radiation resistance, where RHOA inhibition hinders the activity of cancer-related fibroblasts (256). Interestingly, RHOA mutations have also been associated with failure of anti-tumor response and therapy resistance. Thus, Kumagai reported that RHOA tumors (gastric tumors) carry a metabolic advantage, favouring the accumulation of Tregs within the tumor and subsequent immunosuppression, underlying resistance to immune checkpoint therapy (257). Together, this further highlights the clinical relevance of RHOA in the context of cancer.

## 3 Discussion

RHOA is a key regulator of cytoskeletal dynamics, cell adhesion and motility, and thereby controls epithelial homeostasis. In the intestinal epithelium, proper regulation of RHOA is essential for coordinating actomyosin dynamics and tight junction remodelling during cell shedding. Dysregulation, due to either altered prenylation or RHOA deficiency, compromises barrier integrity, increases epithelial alterations, and inflammation (9, 80). Both increased and decreased RHOA activity has been associated with CRC development, highlighting its context-dependent role in tumorigenesis. This underscores the need for precise spatiotemporal regulation of RHOA activity, further complicated by the interaction between RHOA and its regulators (GEFs, GAPs, GDIs) and post-translational modifications that can alter its signalling output. Furthermore, the downstream effects of RHOA activation are further determined by the specificity of the effectors, depending on the cellular context.

It is important to emphasize that the role of RHOA in CRC is influenced by the inflammatory state, which is something to potentially consider to segregate sporadic CRC in the absence of chronic inflammation, and CAC arising upon prolonged and unresolved inflammation, promoting genomic instability and oncogenic mutations (25, 258). Notably, RHOA and other components of the RHO/RAC pathway are frequently mutated in CAC (8), highlighting their relevance as modulators of inflammation-driven tumorigenesis. However, although dysregulation of RHO/RAC signalling may play a functional role in the progression of CAC, the context-dependent and pleiotropic nature of RHOA signalling complicates the identification of effective therapeutic strategies. Of note, replicating and studying human CAC in a mouse model is challenging. CAC typically develops in patients who have been suffering from colitis for about 10 years and have developed mutations gradually over time, resulting in typical flat lesions that are difficult to detect with conventional endoscopy. In contrast, commonly used animal models to mimic IBD-associated CRC, such as AOM/DSS, rely on rapid chemically induced mutagenesis and predominantly generate polypoid lesions more reminiscent of sporadic CRC (22). This discrepancy highlights that it would be important to identify new models mimicking CAC more closely. In this context, the study from Robles et al. set an optimal basis for further detailed studies in order to identify the most suitable models, for instance, by targeting the RHO/RAC pathway. Actually, our studies suggest that chronic inflammation due to RHOA inhibition within IECs can finally result in CAC (9, 195). The availability of new models would, in turn, impact the definition of underlying molecular mechanisms explaining the opposite roles upon activation of the same protein, for instance, RHOA.

Among cellular and molecular mechanisms controlled by RHOA, the failure of EDAC under inflammatory conditions provides a plausible explanation for the context-dependent change in RHOA function, where the same signalling pathway may exert both a tumour suppressor and tumor-promoting effect depending on the tissue microenvironment and inflammatory context. Thus, the chronic inflammatory environment in CAC can compromise EDAC (79). In fact, mice carrying RHOA-deficient epithelium depict intestinal leakage, inflammation and defective cell shedding (9), which can be understood as the sequence of events leading to impaired EDAC resulting in tumor formation. Similarly, mutations affecting RHOA-related pathways, such as APC-dependent alterations of epithelial tension, may further disrupt cellular competition and promote tumour initiation (83, 93). In addition to epithelial cells, RHOA modulates the immune microenvironment, influencing antigen presentation by dendritic cells, macrophage function, T cell differentiation, Treg cell stability, B cell development, and neutrophil motility (136, 142, 259). These functions suggest that RHOA activity can both alter anti-tumour immunity and contribute to inflammation that promotes tumour development, further reinforcing the hypothesis that its effects are context-dependent and can lead to distinct outcomes. This additional complexity should be taken into account when developing therapeutic strategies, since RHOA modulation may simultaneously affect tumour and immune cells.

Overall, although several pharmacological strategies have been developed to modulate RHOA signalling, including direct inhibitors, effector-acting molecules, and prenylation blockers, their clinical translation is limited by the lack of specificity, offset effects and pleiotropic roles of Rho family GTPases. These challenges highlight the need for approaches that can selectively modulate discrete RHOA-dependent pathways, particularly considering the dual role of RHOA and the potential opposite effects observed in inflamed versus non-inflamed tissues. Future studies should therefore focus on analysing the precise molecular contexts, such as the presence of inflammation, specific interactions of regulatory proteins, and post-translational modifications, that determine the pro- or anti-tumour behaviour of RHOA, to inform disease-specific therapeutic strategies for both sporadic CRC and CAC.
